# A case of granulomatosis with polyangiitis complicated by cyclophosphamide toxicity and opportunistic infections: choosing between Scylla and Charybdis

**DOI:** 10.1186/1471-2369-15-28

**Published:** 2014-02-04

**Authors:** Elena Ernst, Matthias Girndt, Rainer U Pliquett

**Affiliations:** 1 Martin-Luther-University Halle-Wittenberg, Clinic of Internal Medicine 2, Department of Nephrology, Ernst-Grube-Str. 40, 06120 Halle (Saale), Germany

**Keywords:** Cyclophosphamide toxicity, Granulomatosis with polyangiitis, Immunosuppression, Opportunistic infections

## Abstract

**Background:**

We report a case of progressive Granulomatosis with Polyangiitis (Wegener’s Granulomatosis) with life-threatening complications of both the underlying disease and induction immunosuppressive therapy. Here, for the first time, cyclophosphamide toxicity and severe opportunistic infections including *pneumocystis jirovecii*- pneumonia were found in one case in a close temporal relationship.

**Case presentation:**

A 34-year-old male patient of Caucasian ethnicity presented with acute renal failure necessitating hemodialysis treatment due to Granulomatosis with Polyangiitis (Wegener’s Granulomatosis). Kidney disease progressed to end-stage renal disease shortly after first diagnosis. After the 2^nd^ bolus of cyclophosphamide shortly, induction immunosuppression (glucocorticoid/cyclophosphamide) was interrupted for repeat infections and resumed 5 years later. By that time, the lungs developed large pulmonary cavernae most likely due to smoldering granuloma indicative for the failed goal of disease remission. Therefore, induction immunosuppression was resumed. Following two monthly boli of cyclophosphamide, the patient developed pericardial effusion and, consecutively, atrioventricular blockade most likely due to cyclophosphamide. After recovery, the patient was discharged without cotrimoxacole. 10 weeks after the last cyclophosphamide bolus and 6 weeks after cessation of cotrimoxacole, the patient was readmitted to the intensive-care unit with Pneumocystis jirovecii pneumonia, and died 6 months later or 74 months after first diagnosis of Granulomatosis with Polyangiitis.

**Conclusions:**

This case illustrates both the need for adequate immunosuppressive therapy to reach disease remission and the limitations thereof in terms of complications including cardiotoxicity of cyclophosphamide and *Pneumocystis jirovecii* pneumonia. In line with current recommendations, the present case strongly encourages *pneumocystis jirovecii*- pneumonia chemoprophylaxis for at least 6 months following induction therapy in Granulomatosis with Polyangiitis.

## Background

Granulomatosis with polyangiitis (GPA), formerly called Wegener’s Granulomatosis, generally presents with pulmonary and renal failure due to vasculitis caused by antineutrophil cytoplasmic antibodies directed against cytosolic proteinase-3 (cANCA). In addition, granuloma formation of the lungs and upper airways may lead to hemoptysis and lung destruction. Less frequently, GPA-associated granuloma occur in the skin, eye, joints, gastrointestinal tract or heart [1-5]. Elevated serum cANCA, clinical clues and/or typical histological findings of kidney biopsies establish the diagnosis of GPA. Cyclophosphamide or monoclonal anti-CD20 antibody (rituximab) therapy is employed as induction immunosuppression to achieve disease remission. Nevertheless, despite optimal induction and maintenance immunosuppression, GPA relapses in about 50% of all cases within five years after first diagnosis, translating into a poorer prognosis [6].

Immunosuppressive therapy renders GPA patients susceptible to life-threatening opportunistic infections such as *pneumocystis jirovecii* pneumonia (PCP). Current recommendations encourage the use of co-trimoxazole as PCP chemoprophylaxis following induction therapy [7,8] as well as in maintenance therapy in GPA [9,10]. Acceptance rate of this recommendation still needs to be improved [11,12]. Here, we report a case of progressive GPA complicated by cyclophosphamide cardiotoxicity and infections including PCP.

## Case presentation

A 34 year-old male patient of Caucasian ethnicity was diagnosed with a 3^rd^ degree acute kidney injury (Acute Kidney Injury Network classification [13]) in a regional German hospital. Co-morbidities included arterial hypertension, obstructive sleep apnea, obesity (body-mass index: 40.4 kg/m^2^), tobacco abuse, and chronic obstructive pulmonary disease (COPD). Based on an elevated serum cANCA titer, the diagnosis of GPA was established. A histological confirmation was obtained by kidney biopsy. Initially, seven sessions of plasmapheresis and induction immunosuppression (500 mg i.v. methylprednisolone for 3 days, steroid taper-off, 0.5 g cyclophosphamide i.v. twice within two months) were applied leading to a partial remission of GPA. However, a bronchoscopy-proven mycotic pneumonia (*aspergillus fumigatus)* occurred following the second bolus of cyclophosphamide requiring antimycotic treatment (voriconacole 200 mg bid) for several months. Immunsuppressive induction therapy was stopped immediately. Hence, due to the halted immunosuppressive therapy, kidney function progressively deteriorated. Eleven months after onset of GPA, the patient had to be hospitalized for hemodialysis-access surgery and hemodialysis initiation. During the following 4 years, hemodialysis-fistula thrombosis and repeat catheter-associated bloodstream infections, episodes of exacerbated COPD and pneumonia occurred. The patient ignored recommendations regarding fluid intake. Because of respiratory symptoms related to overhydration and infections, the patient was deemed unfit to proceed with cyclophosphamide induction. A maintenance therapy with prednisolone (10 mg) was prescribed. Four years after GPA diagnosis, CRP and cANCA serum levels rose, and the patient had to be hospitalized three times within a year due to progressive dyspnea, cough and fever. During the last hospitalization one year before index hospitalization, bronchoscopy ruled out opportunistic infections. Mycophenolate mofetil was introduced as new maintenance immunosuppression for suspected smoldering disease activity in the lungs. Five years after first diagnosis of GPA, the patient received the third and forth intravenous bolus of cyclophosphamide (0.6 g each) within two months. Voriconacole (200 mg twice daily) and co-trimoxazole (960 mg/d) were added as prophylaxis for mycotic pneumonia and PCP. Within less than 24 hours following the 4^th^ cyclophosphamide bolus given after hemodialysis, the patient developed dyspnea at rest necessitating re-hospitalisation, endotracheal intubation and assisted mechanical ventilation. Echocardiography showed a pericardial effusion (end-diastolic: 2.5 cm), massive right-ventricular dilation and hypokinesia. New ECG changes triggered an immediate coronary angiography exam which excluded clogged coronaries. Right-ventricular catheterization showed an elevated pulmonary-artery pressure: 74/54/58 mmHg (systolic/diastolic/mean). There, pulmonary-artery embolism was excluded. Cardiac enzymes remained unrevealing. The pericardial effusion disappeared after one month. As another sign of cyclophosphamide toxicity, anemia and neutropenia occurred. Anemia had to be corrected by two packed erythrocyte concentrates. Neutropenia was complicated by sepsis with elevated procalcitonin (PCT: 13.6 ng/ml) and C-reactive protein (CRP: 259 mg/l) one week after the 4^th^ bolus of cyclophosphamide. The patient was again on assisted mechanical ventilation for 11 days. Thoracic computed tomography showed pulmonary infiltrates within large, partly calcified pulmonary cavernae (maximum 6 cm in diameter). Under intensified antibiosis guided by microbiologic findings from bronchoalveolar lavage, the sepsis subsided. Coincidentally, either GPA disease activity or late sequelae of cyclophosphamide, the patient had to be reanimated for third-degree atrioventricular block two weeks after the 4^th^ bolus of cyclophosphamide. The latter possibility appears to be more likely because cytoplasmic ANCA titer only was 1:20 seven days before. A permanent pacemaker was inserted, and the patient was discharged with p.o. antibiosis, p.o. prednisolone (10 mg/d), however, without voriconacole or co-trimoxazole three weeks after admission. Six weeks following discharge or 10 weeks after the fourth cyclophosphamide bolus, the patient was readmitted for suspected pneumogenic sepsis with worsening dyspnea and hypotension. At admission, the patient had to be transferred to an intensive-care unit (ICU) requiring catecholamine therapy and mechanical ventilation for five days. Blood cultures remained negative. Sputum revealed *Pneumocystis jirovecii*, *Candida albicans*, *Peptostreptococcus micros*, and *Actinomyces israelii.* Tuberculosis was ruled out using blood test and in culture of bronchoalveolar fluid following bronchoscopy. Both *Cytomegalovirus* (CMV) IgM and IgG were positive. However, weekly CMV PCR of serum samples remained negative. Both T-helper lymphocyte count (242/μl) and plasma immunoglobulin G level (6.4 g/l) were low indicative for a state of over-immunosuppression.

**Figure 1:**
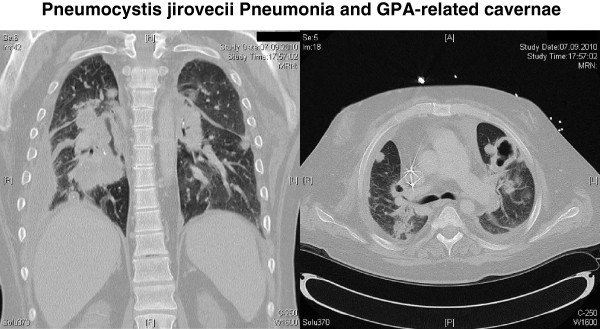
Representative thoracic computed tomography scan at the time of hospitalisation for Pneumocystis jirovecii pneumonia (PCP).

**Figure 2:**
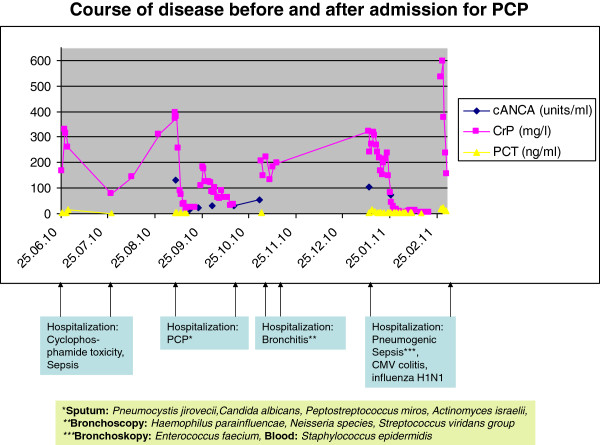
**Time course of C-reactive protein (CRP), procalcitonin (PCT) and anti-neutrophil cytoplasmic antibodies directed against cytosolic proteinase-3 (cANCA) during opportunistic infections (elevated CRP and PCT) and suspected relapses (elevated CRP and cANCA).** The time of hospitalisation is indicated. In addition, relevant microbiologic results were provided.

In addition, a GPA relapse was suspected based on an elevated cANCA concentration (129.9 U/ml). Thoracic computed tomography showed the known pulmonary cavernae and new pulmonary opacities consistent with PCP or GPA-associated vasculitis (Figure 1). Lung-function tests revealed an impaired vital capacity (2.08 l or 54% of reference value), ruled out obstruction (Tiffenau test: 77.7% of maximum vital capacity or 103% of reference value). Intravenous i.v. co-trimoxazole therapy (1 × 1920 mg i.v. for 2 days, 3 × 1920 mg dissolved in 500 ml saline for 12 days) was applied in addition to i.v. antibiosis (imipenem-cilastatin for 14 d) and immunosuppression (prednisolone 50 mg/d). For the suspected recurrent GPA, daily plasmapheresis was conducted over 5 days since admission with 3 liter or 70% of estimated patient’s plasma volume of 5% human-albumin solution. Under daily hemodialysis, the patient’s condition improved, cANCA concentration decreased (29.3 U/ml), serum inflammatory parameters partly normalized (Figure 2). Following discharge (38 days after admission), peroral co-trimoxazole (960 mg/d) and prednisolone (40 mg/d) were maintained. Two weeks later, the patient was readmitted for suspected pneumonia. Laboratory tests revealed an increased CRP (206 mg/l), PCT (2 ng/ml) and cANCA concentration (54.9 U/ml). Computed tomography of the chest was consistent with bronchitis, ruled out pneumonia. Bronchoscopy-guided peroral antibiosis with moxifloxacin (400 mg/d) was initiated, and maintenance immunosuppression (azathioprin 75 mg/d, prednisolone 25 mg/d) was increased for a suspected GPA relapse. However, two months later, azathioprin had to be discontinued, and prednisolone was reduced (10 mg/d) due to an aggravated respiratory infection (CRP 121 mg/l). Body weight (95 kg) was found to be decreased by 33 kg or by 25% since PCP onset. Despite antibiosis, a pneumogenic sepsis occurred. The patient was admitted to an ICU admission, received mechanical ventilation and extracorporal carbon dioxyde elimination. Intravenous antibiosis was adjusted to bronchoalveolar-lavage results. During the 5-months stay at the ICU, a non-ST segment elevation myocardial infarction occurred requiring a percutanous coronary intervention. Shortly thereafter, an intracranial bleeding under anticoagulation with epileptic convulsion, a biopsy-proven cytomegalovirus colitis and viral pneumonitis (H1N1 influenza) complicated the course of disease. Despite several switches of antimicrobial and antiviral therapy, the patient died 74 months after GPA onset.

## Conclusions

This case of GPA illustrates the narrow path between therapeutic control of GPA activity and opportunistic infections or complications due to medical immunosuppression. The overall goal is disease remission without infectious complications or side effects. To reach this goal, various variables including age, renal and liver function need to be considered on an individual basis. Most importantly, a concomitant infection needs to be ruled out prior to medical immunosuppression. In that regard, plasma-exchange therapy may be the initial modality of choice to restore kidney function [14] given its rapid action to reduce antibody load and its moderate immunosuppressive action, especially when the exchange medium used is human plasma.

Here, PCP occurred 8 weeks following cessation of PCP chemoprophylaxis or 10 weeks after the last bolus of cyclophosphamide. From this case, the role for PCP chemoprophylaxis in GPA after induction immunosuppression is emphasized. It is tempting to speculate that an ongoing PCP chemoprophylaxis would have avoided or greatly diminished the risk for PCP in this patient. Clearly, the current practice on PCP chemoprophylaxis in vasculitis such as GPA should be streamlined by guidelines [15] which has been done so far by the European League Against Rheumatism [16]. There, co-trimoxacole (800/160 mg on alternate days or 400/80 mg daily) is recommended. In analogy to kidney transplant recipients [17,18], the time of prophylaxis should cover 6 months after induction immunosuppression in GPA. In addition, PCP chemoprophylaxis may be considered, if cellular immunity is found to be greatly attenuated as indicated by a reduced total lymphocyte count [19], a reduced T-helper lymphocyte count (<400/μl) or the presence of end-stage renal disease [20]. Besides its role in PCP prophylaxis, co-trimoxazole (800/160 mg twice daily) may be considered as an adjunct to standard maintenance therapy with regard to risk reduction of GPA relapse.

Regarding the underlying disease, neither the initial two cyclophosphamide boli nor the third and fourth one five years later led to complete remission of GPA. Shortly after GPA diagnosis, immunosuppressive induction therapy was withheld due to infections. Meanwhile, GPA progressed to end-stage-renal disease and caused massive pulmonary defects that, in turn, became persistent infectious foci. Clearly, disease remission is a prerequisite for an acceptable long-term outcome in GPA, even in the case when end-stage renal disease has been reached. The presence of end-stage renal disease was not shown to slow down GPA progression or prevent relapses of GPA [21]. Besides the need for disease remission, the continued tobacco abuse and the incompliant drinking behaviour leading to over-hydration may have favoured infectious complications in this case as well.

Clinically, infections may have obscured GPA symptoms. To differentiate between infection and GPA progression, regular radiological and laboratory assessments are mandatory. Radiological signs and laboratory parameters including CRP and cANCA may raise suspicion for GPA progression. In addition, as an indirect proof, a set plasmapheresis sessions may help differentiate between GPA progression (responder of plasma exchange) and infection (elevated PCT). Here, plasmapheresis improved the clinical picture at first diagnosis of GPA and later on during hospitalisation for PCP indicating that GPA progression concurrently occurred.

Side effects of immunosuppressive therapy for GPA are another concern. The timely relationship between cyclophosphamide therapy and the occurrence of acute right-heart failure and pericardial effusion suggested cyclophosphamide cardiotoxicity in analogy to previous reports [22-24]. Cyclophosphamide toxicity concerns arise in all patients with renal dysfunction even though the cyclophosphamide dose was relatively low (600 mg) in the present case. To safeguard for toxicity, the timing of hemodialysis (12 hours after cyclophosphamide exposure) is pertinent [25]. This requirement was not fulfilled here.

In summary, this case underlines the importance of disease remission in GPA, and supports the concept of PCP chemoprophylaxis with co-trimoxazole for up to 6 months after intensified medical immunosuppression. Co-trimoxazole as adjunct to maintenance therapy in GPA would serve both as life-long PCP prophylaxis and remission control [26,27]. In addition, the role of novel vasculitis activity markers such as circulating endothelial cells and endothelial microparticles [28] needs to be assessed in clinical medicine to detect relapses early and tailor immunosuppressive therapy accordingly. Together, comprehensive maintenance immunosuppressive therapy, monitoring of (emerging) vasculitis markers and determination of T-helper lymphocyte count may help guide GPA therapy to avoid relapses (Scylla) and over-immunosuppression (Charybdis).

## Consent

Written informed consent for publication of this Case Report and any accompanying images was obtained directly from the patient before his death. A copy of the written consent is available for review by the Editor of this journal.

## Abbreviations

AKIN: Acute kidney injury network classification; cANCA: Antineutrophil cytoplasmic antibodies directed against cytosolic proteinase-3; COPD: Chronic obstructive pulmonary disease; CRP: C-reactive protein; GPA: Granulomatosis with polyangiitis; ICU: Intensive-care unit; PCP: *Pneumocystis jirovecii* pneumonia; PCT: Procalcitonin.

## Competing interests

The authors declare that they have no competing interests.

## Authors’ contributions

EE made substantial contributions to conception, or acquisition of data. MG gave critical input in revising the manuscript. RUP drafted the manuscript. All authors contributed to the content and approved the final version of the manuscript to be published.

## Pre-publication history

The pre-publication history for this paper can be accessed here:

http://www.biomedcentral.com/1471-2369/15/28/prepub
